# Garlic Oil Alleviates MAPKs- and IL-6-mediated Diabetes-related Cardiac Hypertrophy in STZ-induced DM Rats

**DOI:** 10.1093/ecam/neq075

**Published:** 2010-09-15

**Authors:** Sheng-Huang Chang, Chung-Jung Liu, Chia-Hua Kuo, Hong Chen, Wen-Yuan Lin, Kun-Yu Teng, Sheng-Wei Chang, Chang-Hai Tsai, Fuu-Jen Tsai, Chih-Yang Huang, Bor-Show Tzang, Wei-Wen Kuo

**Affiliations:** ^1^Institute of Biochemistry and Biotechnology, Chung Shan Medical University, Taichung, Taiwan; ^2^Tsao-Tun Psychiatric Center, Department of Health, Executive Yuan, Taiwan; ^3^Institute of Basic Medical Science, China Medical University, Taichung, Taiwan; ^4^Division of Environmental Health and Occupational Medicine, National Health Research Institutes, Miaoli County, Taiwan; ^5^Division of Gastroenterology, Department of Internal Medicine, Kaohsiung Medical University Hospital, Kaohsiung, Taiwan; ^6^Laboratory of Exercise Biochemistry, Taipei Physical Education College, Taipei, Taiwan; ^7^Department of Family Medicine, China Medical University and Hospital, Taiwan; ^8^Department of Biological Science and Technology, China Medical University, Taiwan; ^9^Department of Healthcare Administration, Asia University, Taiwan; ^10^Graduate Institute of Chinese Medical Science, China Medical University, Taiwan; ^11^Department of Health and Nutrition Biotechnology, Asia University, Taiwan; ^12^Department of Biochemistry, School of Medicine, Chung Shan Medical University, Taiwan; ^13^Clinical Laboratory, Chung Shan Medical University Hospital, Taichung, Taiwan

## Abstract

Garlic oil has been reported to protect the cardiovascular system; however, the effects and mechanisms behind the cardioprotection of garlic oil on diabetes-induced cardiaomyopathy are unclear. In this study, we used streptozotocin (STZ)-induced diabetic rats to investigate whether garlic oil could protect the heart from diabetes-induced cardiomyopathy. Wistar STZ-induced diabetic rats received garlic oil (0, 10, 50 or 100 mg kg^_1^ body weight) by gastric gavage every 2 days for 16 days. Normal rats without diabetes were used as control. Cardiac contractile dysfunction and cardiac pathologic hypertrophy responses were observed in diabetic rat hearts. Cardiac function was examined using echocardiography. In addition to cardiac hypertrophy-related mitogen-activated protein kinases (MAPK) pathways (e.g., p38, c-Jun N-terminal kinases (JNK) and extracellularly responsive kinase (ERK1/2)), the IL-6/MEK5/ERK5 signaling pathway was greatly activated in the diabetic rat hearts, which contributes to the up-regulation of cardiac pathologic hypertrophy markers including atrial natriuretic peptide (ANP) and B-type natriuretic peptide (BNP), and leads to cardiac contractile dysfunction. Garlic oil treatment significantly inhibited the up-regulation in MAPK (e.g., p38, JNK and ERK1/2) and IL-6/MEK5/ERK5 signaling pathways in the diabetic rat hearts, reducing the levels of cardiac pathologic hypertrophy markers such as ANP and BNP, and improving the cardiac contractile function. Collectively, data from these studies demonstrate that garlic oil shows the potential cardioprotective effects for protecting heart from diabetic cardiomyopathy.

## 1. Introduction

Diabetes mellitus (DM) is one of the major risk factors for cardiovascular disease development, accounting for 80% of all diabetic mortality [1]. In addition, the mortality of cardiac disease in patients with diabetes is 2- to 4-fold higher than that in subjects without diabetes [2]. The destruction of cardiac function has been well documented in both clinical and experimental diabetes [3–5]. Several pathological processes may initiate myocyte injury and myocardial dysfunction in patients with diabetes. Diabetic cardiomyopathy, characterized by cardiac hypertrophy and the presence of diastolic and/or systolic contractile dysfunction, eventually leads to heart failure [6].

Cardiac hypertrophy is defined as an enlargement of the heart with the increase in volume of cardiomyocyte cell. Cardiac hypertrophy is a multi-step process that occurs in response to various pathological stimuli such as myocardial infarction (MI), hypertension, valvular insufficiency, infectious agents and endocrine disorders [7, 8]. Under these stimuli, hypertrophic growth of myocardium is required to adapt to the increased work load of the heart and/or preserve the heart pump function by increasing muscle mass [8, 9]. Cardiac pathological hypertrophy is characterized by an increase in cell size, sarcomere reorganization and activation of the fetal genes including atrial natriuretic peptide (ANP), B-type natriuretic peptide (BNP), *β*-myosin heavy chain and skeletal alpha actin [10, 11]. Prolonged hypertrophic status has been reported to be associated with the decompensation of heart function, the development of heart failure and sudden death in humans [12–14]. Epidemiological studies have showed that cardiac hypertrophy is an independent risk factor for cardiac morbidity and mortality [15, 16].

Cardiac hypertrophy response is typically induced by active membrane-bound receptors that lead to the activation of intracellular signaling pathways through multiple GTPase proteins, kinases and phosphatases. A large number of studies have been dedicated to exploring the molecular mechanisms behind the hypertrophic response of myocardium [17]. Mitogen-activated protein kinases (MAPKs) include three major subfamilies such as the extracellularly responsive kinases (ERKs), the c-Jun N-terminal kinases (JNKs), also known as stress-activated protein kinases (SAPKs), and the p38 MAPKs [18]. In the heart, MAPK signaling pathways have been reported to participate in the development of cardiac hypertrophy in response to stimuli [10, 19, 20]. For example, depletion of ERK1/2 signaling pathway using antisense oligodeoxynucleotides significantly attenuated phenylephrine (PE)-induced cardiomyocyte hypertrophy in culture [21]. MEK1 inhibitor U0126 greatly inhibited both endothelin-1 (ET-1) and PE-induced cardiomyocyte hypertrophy *in vitro* [22]. p38-MAPK was observed in pressure overload [23, 24], and ET-1/PE stimulation [25], and the increased p38-MAPK activity has been showed to involve in many cardiovascular diseases such as hypertension, cardiac hypertrophy, myocardial ischemia and heart failure [26]. In cardiomyocytes, the increased activity of JNK is sufficient to induce cardiomyocyte hypertrophy in response to mechanical stretching [27] or hypertrophic agonists such as ET-1 [28], PE [29] or angiotensin II (Ang II) [30]. Down-regulation of JNK activity by dominant-negative MKK4 mutant or MEKK1 disruption significantly reduced the hypertrophic response induced by ET-1 [28], pressure overloading [31] and ischemia/reperfusion [32]. Elevated intracardiac interleukin-6 (IL-6) is reported and causes cardiac hypertrophy through the IL-6 signal transducing receptor component, glycoprotein 130 (gp130), which eventually leads to heart failure (HF) [33]. The activated MEK5/ERK5 signaling pathway by IL-6 family cytokines, leukemia inhibitory factor (LIF) and carddiotrophin-1 (CT-1) has been shown to result in the serial assembly of sarcomeres and eccentric cardiac hypertrophy [34].

Garlic (*Allium sativum*) has played important dietary and medicinal roles for centuries. Due to its exhibition of inhibiting enzymes involved in lipid synthesis, decreasing platelet aggregation, preventing lipid peroxidation of oxidized erythrocytes and low-density lipoprotein, increasing antioxidant status and inhibiting angiotension-converting enzyme [35–38], today the medicinal use of garlic is widespread and growing. The main property of garlic for therapeutic effects is as an effective antioxidant against oxidative damage in cardiovascular diseases [39]. In addition, according to Ryan's report [40], garlic is also the most commonly used alternative medication by diabetic patients. The antihyperglycemic effect of garlic has been reported in several studies [41–44]. It was also reported that garlic can improve hyperglycemia in diabetic patients [45]. However, information about the effects of garlic on the diabetic heart is very limited.

Therefore, we hypothesize that cardiac contractile dysfunction and cardiac pathologic hypertrophy induced by diabetes can be alleviated by garlic oil supplement. The results demonstrated that garlic oil treatment down-regulates activities of MAPKs (e.g., p38, JNK and ERK1/2) and IL-6/MEK5/ERK5 signaling pathways, and reduces expressions of pathologic hypertrophy response genes such as ANP and BNP, which contributes to the improvement of cardiac contractile function.

## 2. Methods

### 2.1. Garlic Oil Preparation

Garlic oil was prepared as described [46]. Briefly, a steam distillation technique was used, and the final product contained major garlic oil essential components, including diallyl disulfide, diallyl trisulfide, diallyl sulfide and minor amounts of many other volatile compounds.

### 2.2. Animal Model and Treatments

Male Wistar rats (4 weeks old) were purchased from the National Animal Breeding and Research Center (Taipei, Taiwan). The animals were kept under a 12-h light-dark cycle and ambient temperature was maintained at 25°C. Animals were given free access to water and standard laboratory chow (Lab Diet 5001; PMI Nutrition International Inc., Brentwood, MO, USA). Housing conditions and experimental procedures were performed according to the NIH Guide for the Care and Use of Laboratory Animals and all protocols were approved by the Institutional Animal Care and Use Committee of Chung Shan Medical University, Taichung, Taiwan. After 1 week of acclimatization, diabetes was induced by injection of streptozotocin (STZ, 65 mg kg^−1^ body weight (BW) in citrate buffer, pH 4.5) into a lateral tail vein [47]. Control rats were injected with the same volume of citrate buffer. After 3 days of injection, animals in which diabetes had been successfully induced were randomly separated into four groups and fed 10, 50 and 100 mg kg^−1^ BW garlic oil or vehicle (corn oil, 2 ml kg^−1^ BW) for 16 days. It was done by daily oral gavages in the vehicle corn oil. The nondiabetic control animals were fed corn oil (2 ml kg^−1^ BW). After 16 days of treatment, all animals were anesthetized and echocardiography was performed. Then, they were sacrificed and their hearts were removed for further analysis.

### 2.3. In Vivo Cardiac Function

Transthoracic echocardiograms were performed at heart rates (HRs) of 300–450 beats per minute in rats anesthetized with isoflurane mixed with O_2_ at flow rate of 5 psi before and 16 days after the garlic oil feeding by an echo machine (Vivid i, 10S transducer, GE Medical Systems, Milwaukee, WI, USA) using a 4–11 MHz phase-array transducer. M-mode images were obtained in the parasternal long- and short-axis views of the left ventricle. Left ventricular end-diastolic diameter (LVEDD), left ventricular end-systolic diameter (LVESD) and posterior wall thickness (PWT) were determined for five cardiac cycles and averaged. The percentage of LV fractional shortening (FS, %) was calculated as ((LVEDD – LVESD)/LVEDD) × 100 (%). Left ventricular end diastolic volume (LVEDV) and left ventricular end systolic volume (LVESV) were used to calculate ejection fraction (EF, %) = ((LVEDV – LVESV)/LVEDV) × 100 (%). LV mass was calculated as 1.855 × ((LVEDD + PW + intraventricular septal thickness (IVS))^3^ – (LVEDD)) (mg) and cardiac output (CO) was calculated as stroke volume × HR (l min^−1^).

### 2.4. Tissue Extraction

The left ventricle samples were homogenized for protein extract in a PBS buffer (0.14 M NaCl, 3 mM KCl, 1.4 mM KH_2_PO_4_, 14 mM K_2_HPO_4_) at a concentration of 1 mg tissue/10 *μ*l PBS for 5 min. The homogenates were placed on ice for 10 min and then centrifuged at 11 000 g for 30 min. The supernatant were collected and stored at −70°C for further analysis.

### 2.5. Protein Contents

The protein content of cardiac tissue extract was analyzed using the Bradford protein assay [48] using the protein-dye kit (Bio-Rad, Richmond, CA, USA). A commercially available bovine serum albumin (Sigma Chemical, St Louis, MO, USA) was used as a standard solution. Changes in optical density were monitored at 595 nm.

### 2.6. Electrophoresis and Western Blot

SDS-PAGE was carried out as described using 10% polyacrylamide gels. After the electrophoresis of samples at 140 V for 3.5 h, the gels were equilibrated for 15 min in 25 mM Tris-HCl, pH 8.3, containing 192 mM glycine and 20% (V/V) methanol. Electrophoresed proteins were transferred to nitrocellulose paper (Amersham, Hybond-C Extra Supported, 0.45 Micro) using a Hoefer Scientific Instruments Transphor Units at 100 mA for 14 h. Nitrocellulose papers were incubated for 2 h in blocking buffer containing 100 mM Tris-HCl, pH7.5, 0.9% (w/v) NaCl, 0.1% (v/v) fetal bovine serum. Monoclonal antibodies (Transduction Laboratories) were diluted (1 : 1000) in buffer containing 100 mM Tris-HCl, pH 7.5, 0.9% (w/v) NaCl, 0.1% (v/v) Tween-20 and 1% (v/v) fetal bovine serum. Incubations were performed for 3.5 h. The immunoblots were washed three times in blotting buffer for 10 min and then immersed in the second antibody solution containing alkaline phosphatase goat anti-rabbit immunoglobulin G (dilution 1 : 2000) (Promega) for 1 h. The filters were then washed three times in blotting buffer for 10 min. Color development was presented in a 20 ml mixture consisting of 7 mg nitro blue tetrazolium, 5 mg 5-bromo-4-chloro-3-indolyl-phosphate, 100 mM NaCl and 5 mM MgCl_2_ in 100 mM Tris-HCl, pH 9.5. Signal intensity was quantitated using a PhosphoImager. *α*-Tubulin was used as a loading control.

### 2.7. Statistical Analysis

Statistical differences were examined by one-way analysis of variance. *P* < .05 was considered statistically significant. Data were expressed as mean ± SE.

## 3. Results

### 3.1. Improvement in Cardiac Contractile Dysfunction in Diabetic Rats in Response to Garlic Oil Feeding

**Figure 1:**
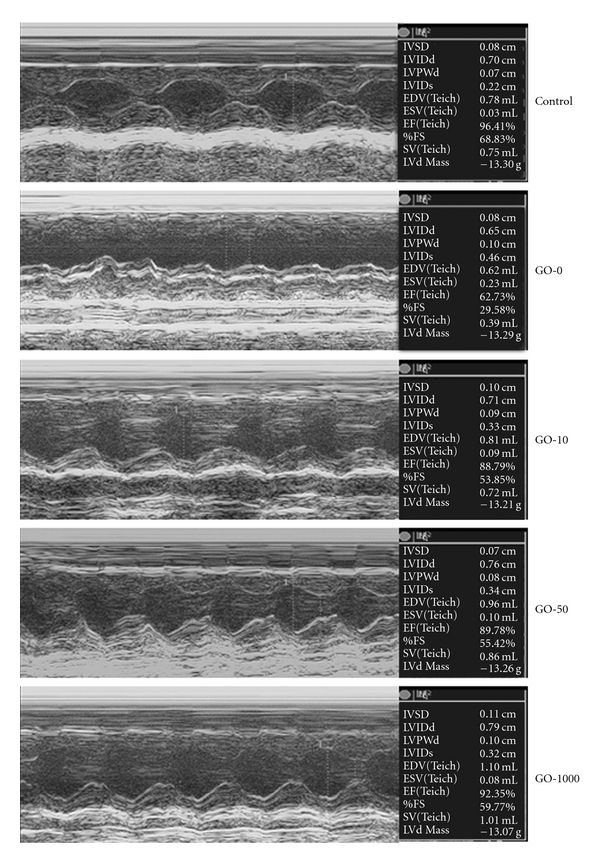
Representative echocardiographic M-mode images from at least four cardiac contractile cycles of control and diabetic rat hearts. GO-0, -10, -50 and -100 represent the doses of 0, 10, 50 and 100 mg garlic oil per kg BW.

In order to assess cardiac function and dimension *in vivo*, we performed echocardiography on diabetic and normal control rats. In addition to significantly high blood glucose induced in diabetic rats, all of the animals with or without STZ-induced diabetes showed a normal appearance and had a normal global cardiac structure and function at Day 0 of induction (Table 1). After 16 days of induction, compared to control group blood glucose increased much more higher and the BW, HR and CO decreased in all diabetic animal groups. The percentage of FS and EF, calculated from the LVEDD or LVEDV and LVESD or LVESV, typically representing cardiac contractile function in rat hearts were largely decreased in diabetic rat hearts. Garlic oil supplement increased HR, CO, FS% and EF% in a dose-dependent manner in diabetic hearts. The BW and blood glucose exhibited unchanged after garlic oil treatment between the four groups of diabetic rats.  Figure 1 shows the echocardiologic M-mode of left ventricular dimensions from rat hearts in both control and diabetic rats fed with different doses of garlic oil. Collectively, garlic oil attenuates the impaired cardiac contractility, and decreased CO and HR in diabetic rat hearts. 

The degree of cardiac hypertrophy in diabetic rats was assessed using the ratio of left ventricle mass (LVM) to tibia length (TL). Although the degree of hypertrophy was higher in diabetic rats than in control rats, the ratio of LVM to TL did not differ between the four groups of diabetic rats.

### 3.2. The Activation of Cardiac Hypertrophy-related MAPKs (p38, JNK and ERK1/2) in the Diabetic Rat Hearts was Reduced by Garlic Oil Supplement

**Figure 2:**
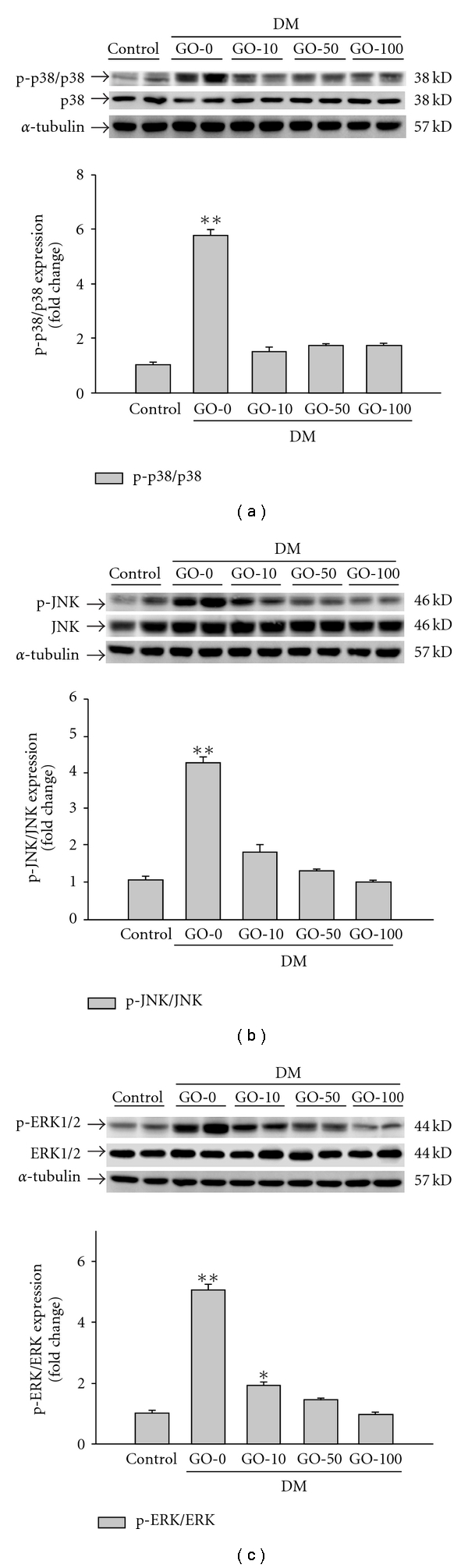
Detection of (a) phosphorylated p38, (b) phosphorylated JNK and (c) phosphorylated ERK1/2 protein levels from the left ventricles in control and diabetic rats, analyzed by western blotting. GO-0, -10, -50 and -100 represent the doses of 0, 10, 50 and 100 mg garlic oil per kg BW. Signal intensity was quantitated using a PhosphoImager. Equal loading was assessed with an anti *α*-tubulin antibody. The average result ± SE of three independent experiments is shown. *P < .05, **P < .01 represent significant differences from control group.

To further identify which signal transduction pathway(s) was involved in the mechanism behind diabetes-induced cardiac hypertrophy, we detected the phosphorylation/activation of cardiac hypertrophy-related MAPKs including p38, JNK and ERK1/2 in left ventricle from the diabetic rat hearts. We found that phosphorylated p38 is significantly increased by *∼*5.74-fold; phosphorylated JNK is significantly increased by *∼*4.32-fold; phosphorylated ERK1/2 is significantly increased by *∼*5.03-fold (Figure 2). Interestingly, the diabetes-induced activation of MAPKs (e.g., p38, JNK and ERK1/2) in the diabetic rat hearts was notably reduced by garlic oil supplement (10, 50 and 100 mg kg^−1^ BW). 

### 3.3. The Up-regulation of Intracardiac IL-6 Expression in the Diabetic Rat Hearts was Reduced by Garlic Oil Supplement

**Figure 3:**
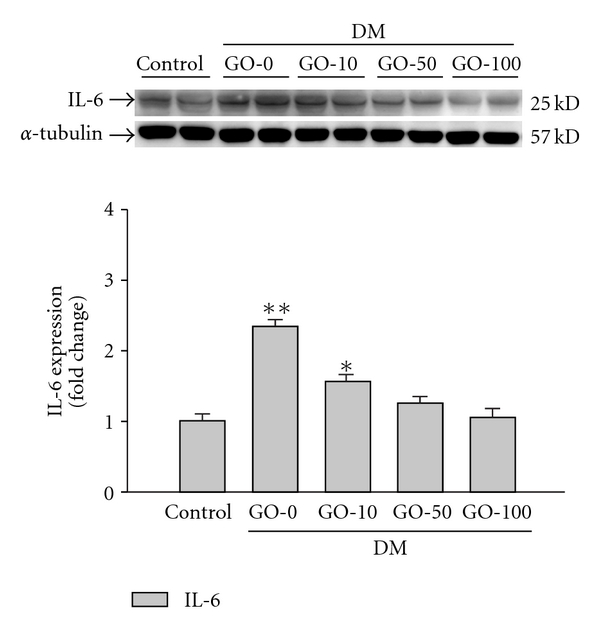
Detection of IL-6 protein levels from the left ventricles in control and diabetic rats, analyzed by western blotting. GO-0, -10, -50 -and -100 represent the doses of 0, 10, 50 and 100 mg garlic oil per kg BW. Signal intensity was quantitated using a PhosphoImager. Equal loading was assessed with an anti *α*-tubulin antibody. The average result ± SE of three independent experiments is shown. *P < .05, **P < .01 represent significant differences from control group.

Overexpression of IL-6 in the myocardium in acute MI (AMI) appears to be associated with the mechanism of cardiac hypertrophy [49]. The left ventricles from diabetic rat hearts showed a significant protein level of IL-6, compared to the control group (Figure 3). On supplementing garlic oil (10, 50 and 100 mg kg^−1^ BW) to the diabetic rats, we found that intracardiac IL-6 expression was reduced in a dose-dependent manner. 

### 3.4. Garlic Oil Supplement Reduces Expression of MEK5 and ERK5 in the Diabetic Rat Hearts

**Figure 4:**
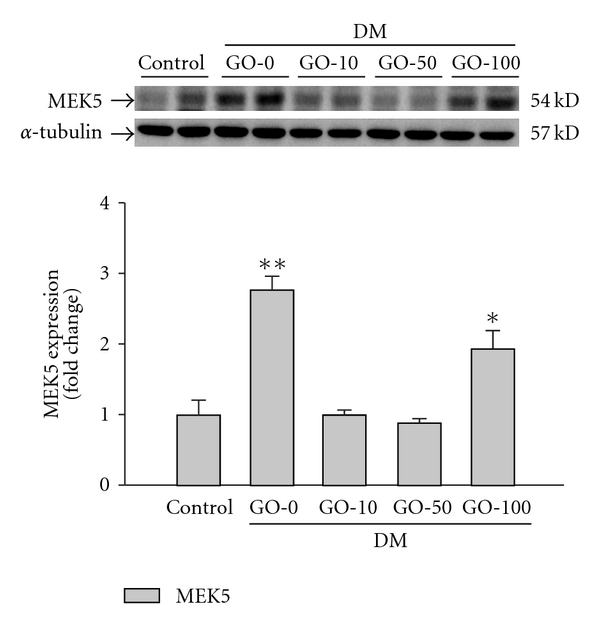
Detection of MEK5 protein levels from the left ventricles in control and diabetic rats, analyzed by western blotting. GO-0, -10, -50 and -100 represent the doses of 0, 10, 50 and 100 mg garlic oil per kg BW. Signal intensity was quantitated using a PhosphoImager. Equal loading was assessed with an anti *α*-tubulin antibody. The average result ± SE of three independent experiments is shown. *P < .05, **P < .01 represent significant differences from control group.

**Figure 5:**
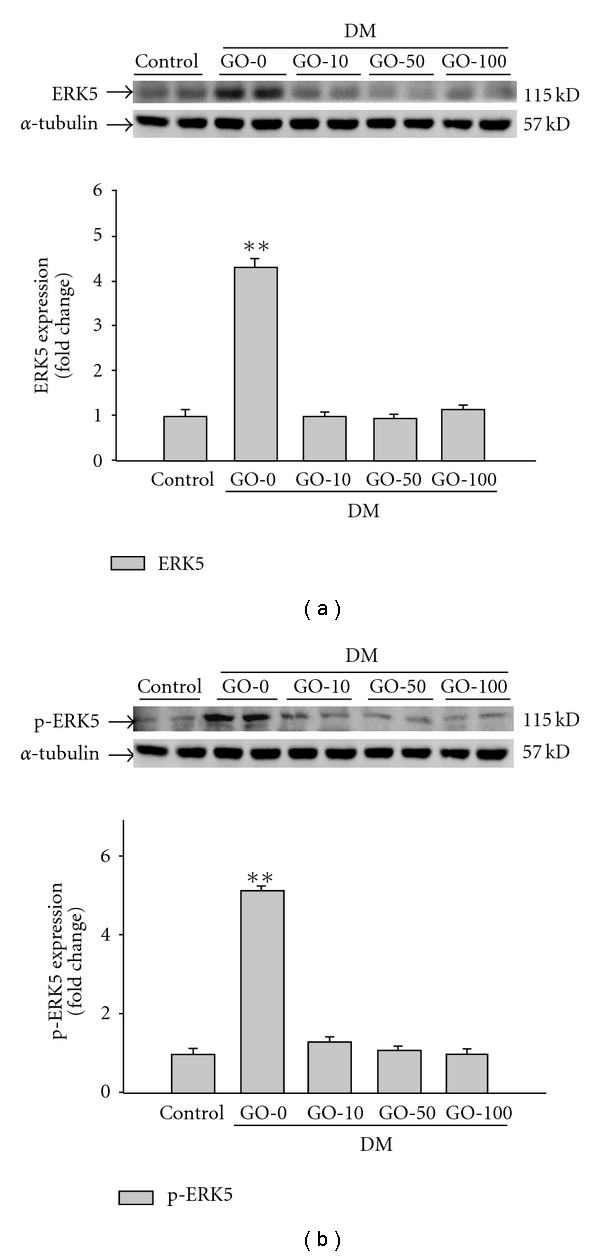
Detection of (a) ERK5 and (b) phosphorylated ERK5 protein levels from the left ventricles in control and diabetic rats, analyzed by western blotting. GO-0, -10, -50 and -100 represent the doses of 0, 10, 50 and 100 mg garlic oil per kg BW. Signal intensity was quantitated using a PhosphoImager. Equal loading was assessed with an anti *α*-tubulin antibody. The average result ± SE of three independent experiments is shown. **P < .01 represent significant differences from control group.

Activated MEK5/ERK5 signaling pathway by IL-6 family cytokines has been reported to lead to the development of eccentric cardiac hypertrophy [34]. In this study, overexpression of MEK5 was detected in the diabetic rat hearts (Figure 4). The downstream ERK5 was also significantly up-regulated in both protein expression (Figure 5(a)) and protein activation (Figure 5(b)) in the diabetic rat hearts. The results suggested that the activated MEK5/ERK5 signaling pathway participates in the development of diabetic cardiomyopathy. The up-regulation in both MEK5 and ERK5 was significantly decreased by supplement garlic oil (10, 50 and 100 mg kg^−1^ BW). 

### 3.5. Pathologic Hypertrophy Marker ANP and BNP in the Diabetic Rat Left Ventricle was Reduced by Garlic Oil Supplement

**Figure 6:**
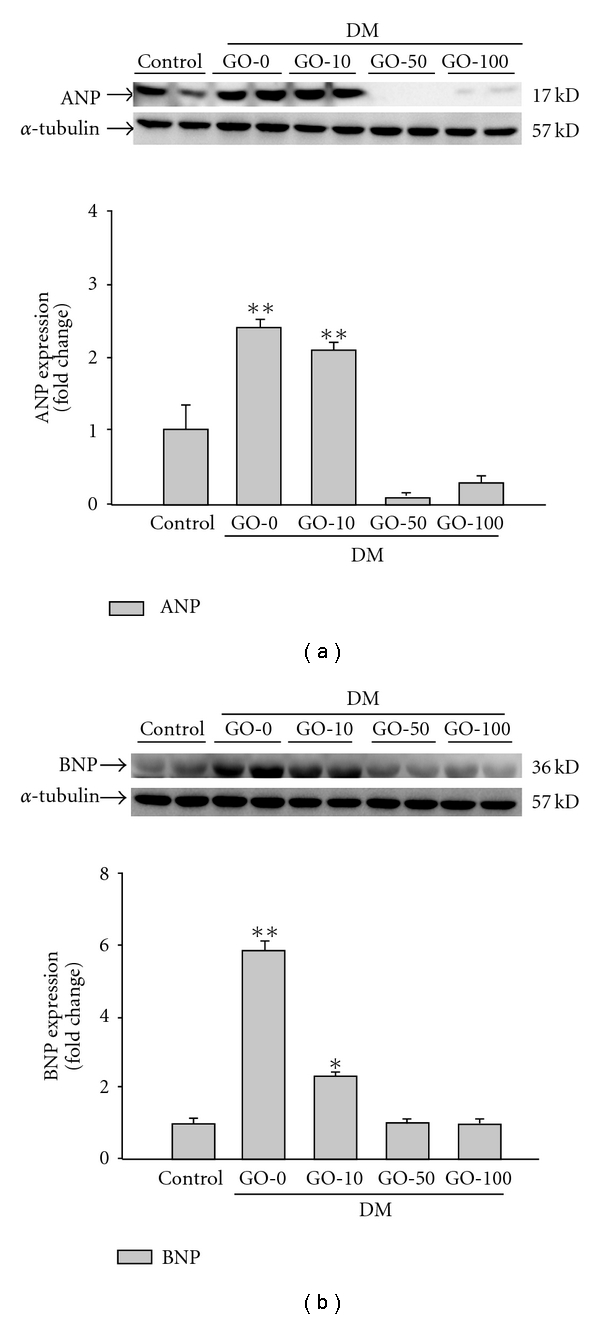
Detection of pathologic hypertrophy marker (a) ANP and (b) BNP protein level from the left ventricles in control and diabetic rats, analyzed by western blotting. GO-0, -10, -50 and -100 represent the doses of 0, 10, 50 and 100 mg garlic oil per kg BW. Signal intensity was quantitated using a PhosphoImager. Equal loading was assessed with an anti *α*-tubulin antibody. The average result ± SE of three independent experiments is shown. *P < .05, **P < .01 represent significant differences from control group.

To further understand whether the expression of proteins associated with cardiac pathologic hypertrophy was reduced by garlic oil treatment in the diabetic rat hearts, the pathologic hypertrophy markers such as ANP and BNP were analyzed by western blot (Figure 6). The left ventricle obtained from the diabetic rat hearts showed the significant expression of ANP (Figure 6(a)) and BNP (Figure 6(b)); however, the up-regulation in ANP and BNP in the hearts of diabetic rats was efficiently reduced by garlic oil supplement (10, 50 and 100 mg kg^−1^ BW). The results suggested that garlic oil significantly reduces the diabetes-induced characteristics of cardiac pathologic hypertrophy, improving the cardiac contractile function in the diabetic rats (Figure 1 and Table 1). 

## 4. Discussion

**Figure 7:**
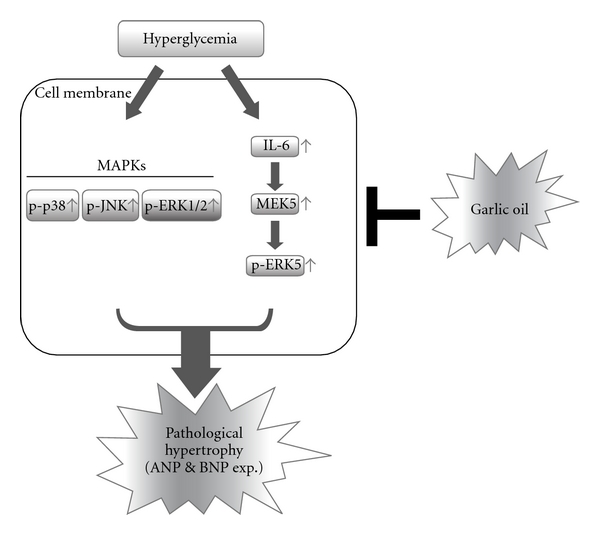
A schematic representation showing garlic oil ameliorates cardiac pathologic hypertrophy and subsequent cardiac dysfunction in rats with diabetes by down-regulating the activities of MAPKs (p38, JNK and ERK1/2) and IL-6/MEK5/ERK5 signaling pathways. Hyperglycemia greatly activates signaling pathways of MAPK (e.g., p38, JNK and ERK1/2) and IL-6/MEK5/ERK5, simultaneously, in the hearts of diabetic rats. These activated signaling pathways significantly induce the development of cardiac pathologic hypertrophy and the expressions of pathologic hypertrophy response genes such as ANP and BNP. In diabetic rats, treatment involving garlic oil reduces the hyperglycemia-cardiac pathologic hypertrophy and improves the heart function by efficiently de-activating MAPK (p38, JNK and ERK1/2) and IL-6/MEK5/ERK5 signaling pathways.

The major findings of the present study can be summarized as follows: (i) STZ-induced diabetes leads to a decrease in HR, cardiac contractile function, CO and contractile velocity in the cardiac muscle. Interestingly, all of these cardiac abnormalities induced by diabetes are improved by garlic oil treatment. (ii) Diabetic cardiomyopathy is structurally characterized by cardiac hypertrophy; in this study, we further identify molecular mechanisms involved in the diabetes-induced cardiac pathologic hypertrophy. Cardiac hypertrophy-related MAPKs (e.g., p38, JNK and ERK1/2) and IL-6/MEK5/ERK5 signaling pathways were greatly activated, which resulted in the expression of pathologic hypertrophy response markers such as ANP and BNP in the heart of diabetic rats. (iii) However, up-regulated activity in MAPKs (p38, JNK and ERK1/2), IL-6/MEK5/ERK5 signaling cascade and the expression of pathologic hypertrophy markers (ANP and BNP) were significantly inhibited when supplying garlic oil (10, 50 and 100 mg kg^−1^ BW). The results suggest that garlic oil possesses the potential cardioprotective effects for heart from diabetic cardiomyopathy (Figure 7). 

Recent data from the Acute Decompensated Heart Failure National Registry demonstrates that *∼*44% of patients with decompensated heart failure suffer from DM [50]. Pathophysiology of DM includes hypoinsulinemia, hyperglycemia, cardiac hypertrophy and a cardiomyopathy that is characterized by the presence of diastolic and/or systolic contractile dysfunction. Prolonged cardiac hypertrophy has been reported to be associated with the decompensation of heart function, the development of heart failure and sudden death in humans [12–14, 51]. Because of the side effects of Western drugs, a large number of studies have focused on the effects of natural products on cardiovascular protection. Various oriental herb extracts or dietary supplement have been adopted for preventing cardiovascular abnormalities or disorders, for example, Li-Fu Formula for reducing cardiac apoptosis [51]; Buyang Huanwu Decoction for protection from ischemic heart disease [52]; fermented wheat germ extract (Avemar) for attenuating chronic hypertension, diabetes or metabolic syndrome-induced cardiovascular symptoms [53]; Tanshinone IIA for treatment of cardiovascular disorders [54]; and garlic extract for improving cardiac functions [55–57]. Garlic extract treatment shows some beneficial effects on modulating the HR, rhythm and force of cardiac contraction [55]. Garlic supplement significantly reduced HR during peak exercise and work load upon the heart, resulting in better exercise tolerance in patients with coronary artery disease [56, 58]. Garlic has a significant blood-pressure-lowering effect by reducing the activity of angiotensin-converting enzyme [57] and has protective effects on blocking hypoxic pulmonary hypertension *in vivo* and attenuating age-related increases in aortic stiffness [59]. In this study, we successfully used the STZ-induced diabetic rats to mimic diabetic human with features of cardiac dysfunction, for example, a decrease in FS and EF [60], and an increase in LVESD [61]. STZ-induced diabetes leads to a decrease in cardiac contractile function, CO and contractile velocity in the cardiac muscle. In this STZ-induced diabetic rat model, we observed that the up-regulation of cardiac pathologic hypertrophy markers ANP and BNP in the diabetic rat hearts was significantly inhibited by garlic oil supplement (10, 50 and 100 mg kg^−1^ BW). Garlic oil supplement indeed improves HR, cardiac contractile function, CO and contractile velocity in the diabetic rat hearts. The results suggest that garlic oil improves cardiac function of the diabetic rats by alleviating the development of cardiac hypertrophy. In the clinical study, the evaluation of garlic oil effect on cardiac protection in diabetic patients is proceeding to confirm the present study.

Evidences show that the stimulation of cardiomyocyte hypertrophic growth like other environmental stresses such as osmotic stress, DNA damage and ultraviolet radiation results in the activation of protein kinase cascades, which in turn activate MAPKs such as p38 MAPK, JNK and EKR1/2 [62]. These serine-threonine kinases have been shown to phosphorylate the important downstream mediators that participate in cellular regulations such as cell proliferation, cell differentiation and cell growth process including cardiac hypertrophy [20, 21, 23, 26, 28, 31, 32, 63]. In the present study, we found that increased phosphorylation/activation of p38, JNK and ERK1/2 was detected in the left ventricle of the diabetic rat hearts. The findings suggest that increased activity of MAPKs such as p38, JNK and ERK1/2 participates in the cardiac pathologic hypertrophy in diabetic rat hearts.

Overexpressed IL-6 in the myocardium as a result of AMI appears to play a central role in pathogenesis of cardiac hypertrophy [33]. Activated MEK5/ERK5 signaling pathway by IL-6 family cytokines has been reported to lead to the eccentric cardiac hypertrophy that progresses to dilated cardiomyopathy and sudden death [34, 64]. In the present study, the left ventricles from diabetic rat hearts showed a significant up-regulation of IL-6, MEK5 and ERK5, suggesting that activated IL-6/MEK5/ERK5 signaling cascade participate in the diabetes-induced pathologic hypertrophy. We further found that the activated MAPKs (e.g., p38, JNK and ERK1/2) and IL-6/MEK5/ERK5 signaling pathway in the diabetic rat hearts were significantly inhibited when applying garlic oil supplement. These findings suggest that garlic oil improves heart function by greatly modifying the intracardiac activity of MAPKs (e.g., p38, JNK and ERK1/2) and IL-6/MEK5/ERK5 signaling pathways.

However, in the present study we also found that MEK5 protein in left ventricle was increased when STZ-induced DM rats were treated with garlic oil (100 mg kg^−1^ BW; GO-100). We speculated that garlic oil treatment more than GO-100 dosage may initiate some side effect in left ventricle. More future studies are needed to investigate the side effect in myocardial cells treated with over-dosage of garlic oil. In the present study, we found that garlic oil treatment improves heart function without significant recovery from BW and the level of blood glucose in STZ-induced DM rats. Besides antioxidative [43, 65] and antiglycative [65] properties, we speculated that survival factors in cardiac cells of left ventricle are up-regulated by garlic oil treatment to protect heart from hyperglycemia-induced injury in STZ-induced DM rats. We will further examine the effect of garlic oil on expression of survival factors in myocardial cells.

In conclusion, our findings show that garlic oil supplement to diabetic hearts leads to several alterations at multiple levels, including the improvement of cardiac contractile functions, the down-regulation of cardiac-hypertrophy-related signaling activities and the decrease in expression of cardiac pathologic hypertrophy response genes ANP and BNP. Garlic oil indeed shows the cardioprotective potential for heart from diabetic cardiomyopathy.

## Funding

National Science Council of Republic of China (grant NSC96-2320-B-039-035-MY3). The authors acknowledge the support of the China Medical University (CMU95-176). This study is also supported by Taiwan Department of Health Clinical Trial and Research Center of Excellence (DOH99-TD-B-111-004) and in part by Taiwan Department of Health Cancer Research Center of Excellence (DOH99-TD-C-111-005).

## Figures and Tables

**Table 1:** Physiological and echocardiographic parameters in rat hearts.

	Control	DM			
		GO-0	GO-10	GO-50	GO-100
At basal level					
BW (g)	189.1 ± 14.4	153.0 ± 14.1*	138.5 ± 17.5*	165.0 ± 15.0*	154.5 ± 11.5*
Blood glucose (mg dl^−1^)	73.8 ± 6.5	269.2 ± 59.8*	215.0 ± 42.3*	256.8 ± 44.5*	218.3 ± 49.5*
HR (beats/min)	415.7 ± 10.5	400.0 ± 22.5	397.4 ± 29.0	418.8 ± 39.1	392.4 ± 21.3
LVEDD (mm)	5.70 ± 0.54	5.29 ± 0.38	6.10 ± 0.75	5.36 ± 0.43	6.07 ± 0.62
LVESD (mm)	2.60 ± 0.29	2.61 ± 0.03	2.95 ± 0.51	2.68 ± 0.29	3.11 ± 0.28
FS (%)	49.8 ± 2.0	48.6 ± 3.5	49.3 ± 4.0	48.0 ± 2.1	49.6 ± 3.9
EF (%)	87.6 ± 0.9	86.7 ± 2.9	86.7 ± 3.5	87.4 ± 1.3	85.6 ± 3.0
CO (l min^−1^)	0.20 ± 0.05	0.19 ± 0.08	0.20 ± 0.06	0.19 ± 0.02	0.19 ± 0.01
LVM (mg)	13.22 ± 0.05	13.29 ± 0.01	13.55 ± 0.03	13.42 ± 0.02	13.50 ± 0.07
After 16-day feeding of different doses of garlic oil					
BW (g)	331.3 ± 10.5	225.0 ± 14.4*	155.0 ± 20.2*	202.5 ± 30.3*	212.5 ± 21.7*
Blood glucose (mg dl^−1^)	96.0 ± 7.4	412.0 ± 12.7*	423.5 ± 7.8*	467.5 ± 19.3*	453.8 ± 18.3*
HR (beats/min)	413.5 ± 1.4	279.0 ± 10.2*	323.0 ± 1.6*	364.5 ± 26.8^#^	439.7 ± 13.6^#^
LVEDD (mm)	6.65 ± 0.2	6.93 ± 0.23	7.70 ± 0.17*	7.45 ± 0.29	7.65 ± 0.29*
LVESD (mm)	2.45 ± 0.14	4.60 ± 0.01*	4.65 ± 0.29*	4.15 ± 0.87^∗,#^	3.30 ± 0.58^∗,#^
FS (%)	62.6 ± 3.6	33.5 ± 2.3*	39.4 ± 0.8*	44.2 ± 0.9*	57.6 ± 1.3^#^
EF (%)	93.4 ± 1.7	68.0 ± 3.0*	75.6 ± 0.8*	80.5 ± 0.9^∗,#^	90.8 ± 0.8^#^
CO (l min^−1^)	0.15 ± 0.66	0.13 ± 0.38	0.25 ± 0.17	0.23 ± 0.01	0.37 ± 0.03^∗,#^
LVM (mg)	13.27 ± 0.17	13.21 ± 0.04	13.21 ± 0.04	13.17 ± 0.01	13.16 ± 0.06
LVM/TL (mg mm^−1^)	0.329 ± 0.001	0.413 ± 0.009*	0.358 ± 0.019	0.343 ± 0.013^#^	0.323 ± 0.002^#^

Results are mean ± SEM.

*P < .05 versus with control group. ^#^ *P* < .05 versus with GO-0 group (*n* = 6 rats in each group).
